# New records of Spotted Bass, *Micropterus punctulatus*, within the Mississippi River Basin, Illinois

**DOI:** 10.1002/ece3.9777

**Published:** 2023-01-24

**Authors:** Andrya L. Whitten, Brandon S. Harris, Jason A. DeBoer, Nerissa N. McClelland, James T. Lamer

**Affiliations:** ^1^ Illinois River Biological Station, Illinois Natural History Survey University of Illinois Urbana‐Champaign Havana Illinois USA; ^2^ Illinois Department of Natural Resources‐Division of Fisheries Havana Illinois USA

**Keywords:** Centrarchidae, distribution, Illinois Waterway, long‐term monitoring, sportfish

## Abstract

Spotted Bass *Micropterus punctulatus*, like many sport fishes, have experienced range expansion through intentional introductions (i.e., legal stocking and illegal transfers) and migration across the United States. In Illinois, USA, native populations of Spotted Bass occur along the eastern and southern border of the state. We report new records of Spotted Bass in their non‐native range of the Illinois Waterway and the Illinois portion of the Upper Mississippi River in addition to collections in their native range in the Illinois sections of the Ohio and Wabash rivers to better understand their current distribution. Continuous, collaborative efforts to track the distribution and expansion of non‐native fishes are important for maintaining and establishing native and non‐native fisheries management objectives and education, as non‐native fishes can influence native species population distribution and dynamics.

## Graphical abstract

On October 1, 2021, an adult Spotted Bass was collected near Chillicothe, Illinois, USA. This is the first recorded Spotted Bass in the Peoria Pool and the second collected in the Illinois Waterway. This collection occurred 51 km upstream of their first documentation.

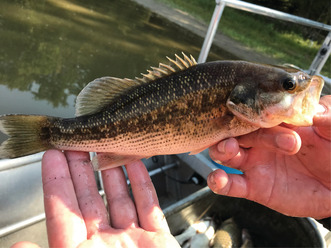

## 1 INTRODUCTION

**FIGURE 1:**
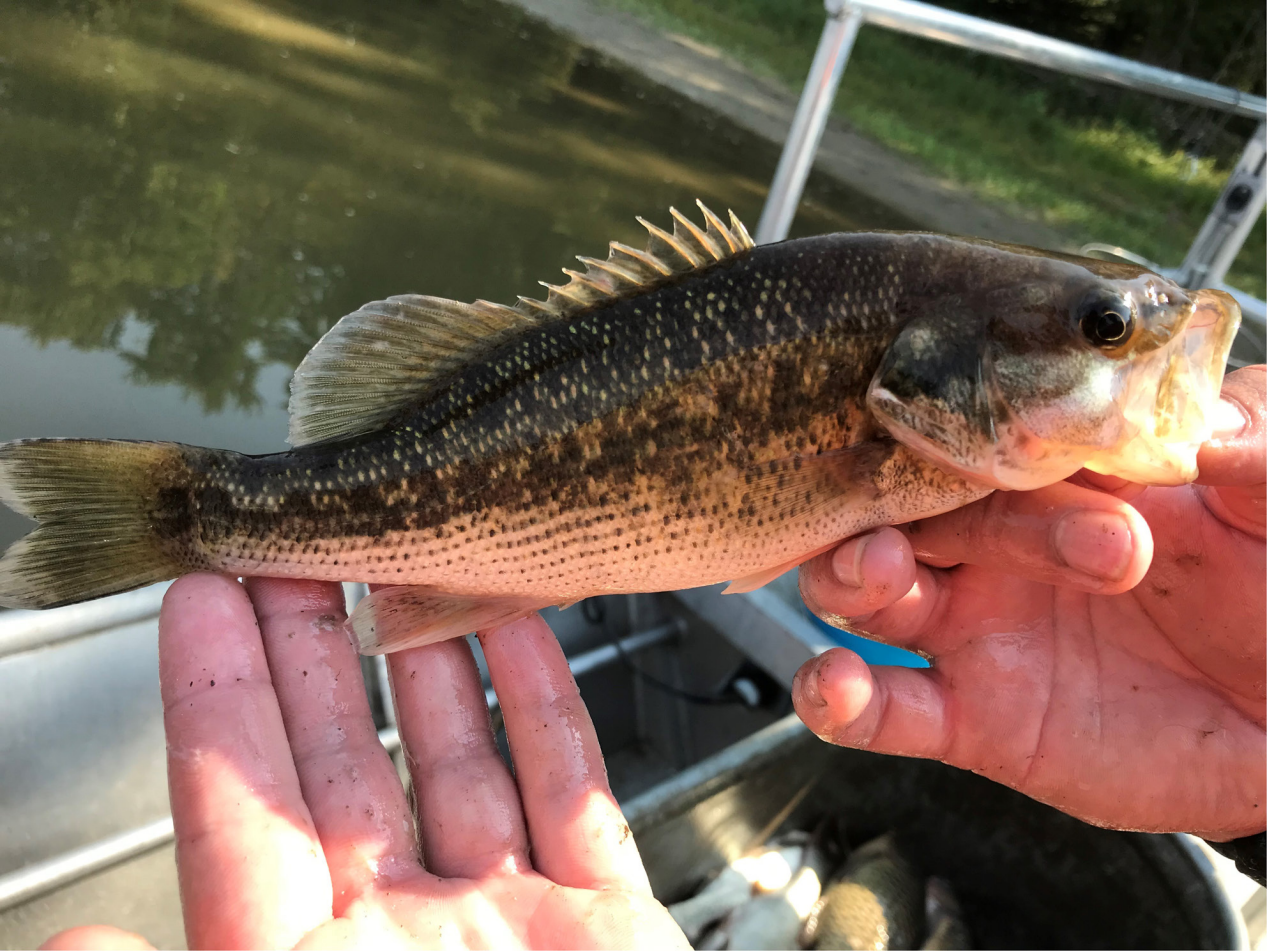
Spotted Bass *Micropterus punctulatus* collected from the Illinois Waterway in 2021. Photograph taken by Andrya L. Whitten.

**FIGURE 2:**
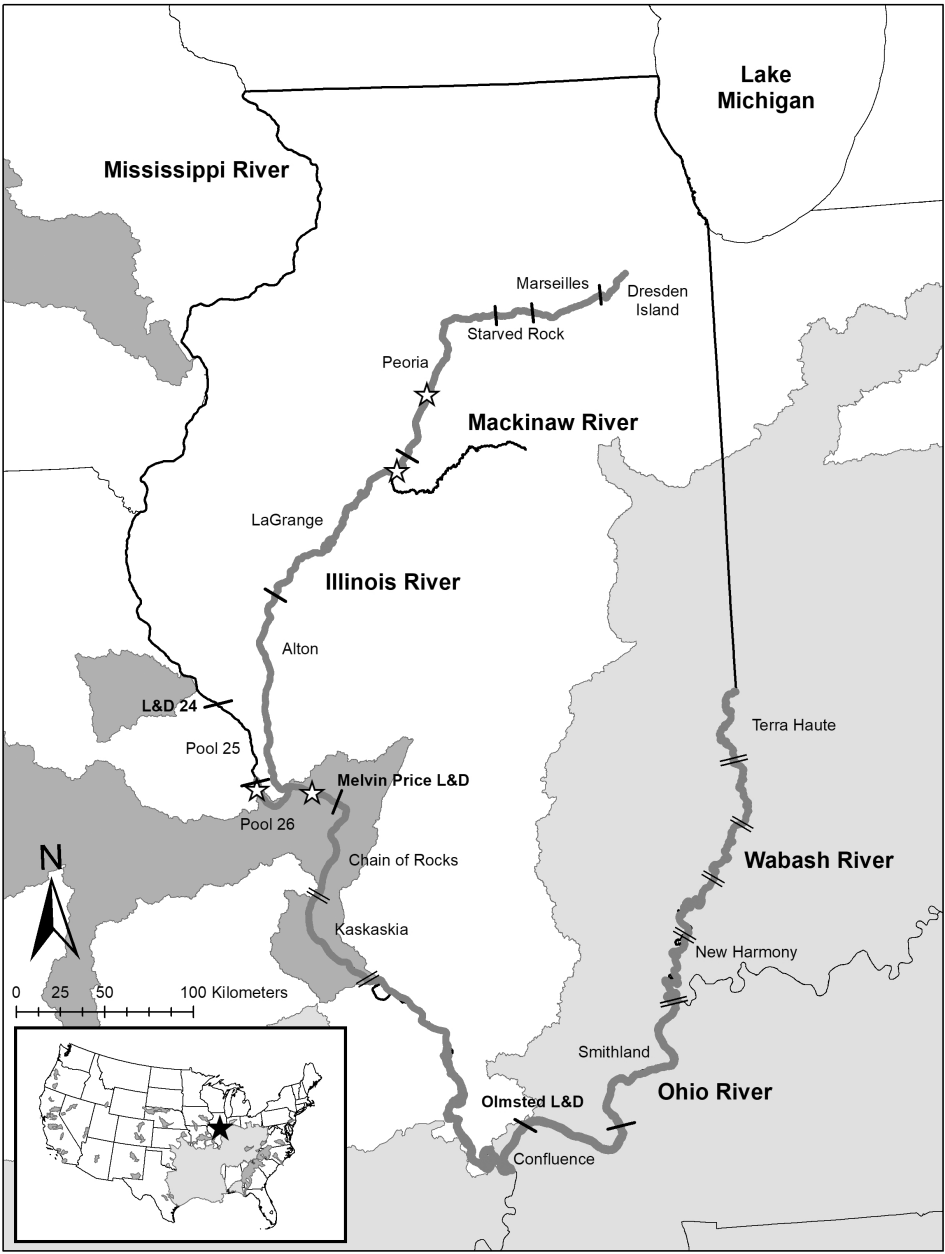
Illinois Waterway and sections of the Upper Mississippi, Ohio, and Wabash rivers sampled by LTEF, LTRM, and IDNR, with the four new Spotted Bass *Micropterus punctulatus* records (2011–2021) denoted by white stars in labeled navigation pools separated by single black bars (lock and dams) in the Illinois Waterway and Upper Mississippi River. Double black bars represent sampled river reaches not separated by lock and dams. The native range of Spotted Bass for the United States and Illinois is represented by light gray shading, and the non‐native range where known populations of Spotted Bass exist is denoted by dark gray shading.

Spotted Bass *Micropterus punctulatus* (Rafinesque, 1819) are a recreationally important sport fish species in reservoirs, streams, and large rivers east of the Rocky Mountains in the South‐Central and Midwestern USA (Pflieger, 1997; Smith, 2002; Figure 1). In Illinois, native, lotic populations of Spotted Bass exist along the eastern and southern border within the Wabash and Ohio rivers and their tributaries (Fuller et al., 2021), and non‐native populations occur along the western border in the Upper Mississippi River (UMR; Figure 2). Although listed as common by the state of Illinois, some conservation programs, such as NatureServe, consider native Illinois populations of Spotted Bass “vulnerable/apparently secure” and at moderate risk of extirpation. This is likely due to their fairly restricted range in Illinois compared with the surrounding states of Indiana and Kentucky, where Spotted Bass populations are listed as “apparently secure” and “secure” (NatureServe, 2022). Widespread range expansion and records of individual occurrences of Spotted Bass throughout the United States have been due to both intentional introductions (i.e., legal stocking and illegal transfers; Churchill & Bettoli, 2015) and natural migration. Tracking the dispersal of non‐native Spotted Bass populations is key to identifying and understanding their potential influence on native species population distribution, dynamics, and management (e.g., Cook et al., 2016), as their effect on native riverine fishes is currently unknown. Thus, here we report four total new collections of Spotted Bass in the Illinois Waterway (IWW) and UMR and document the known collections of Spotted Bass in the Illinois sections of the Upper Mississippi, Ohio, and Wabash rivers through data compiled from three separate monitoring programs.

## 2 METHODS

Spotted Bass collections were compiled for four large rivers in Illinois, the entire IWW and the Illinois portions of the Upper Mississippi, Ohio, and Wabash rivers (Figure 2). The IWW is a large, navigable tributary to the UMR that runs through the interior of Illinois and consists of the Des Plaines and Illinois rivers (Delong, 2005). Illinois is bordered to the west by the UMR that consists of 16 navigational lock and dams, to the south by the Ohio River with two lock and dams, and to the southeast by the unimpounded lower Wabash River (Figure 2). Spotted Bass collections for these four rivers were aggregated from annual monitoring data by the Illinois Department of Natural Resources (IDNR), the U.S. Army Corps of Engineers' Upper Mississippi River Restoration Program's Long‐Term Resource Monitoring (LTRM) element, and the Long‐term Survey and Assessment of Large River Fishes in Illinois (LTEF) program (administered through the University of Illinois, Eastern Illinois University, and Southern Illinois University). Collectively in Illinois, these programs annually sample every pool of the IWW, Pools 12–26 and two open‐river sections of the UMR, the Ohio River from its confluence with the Wabash River to its confluence with the UMR, and the unimpounded lower Wabash River from Terra Haute, IN to New Harmony, IN. These programs use multiple gear types (e.g., daytime electrofishing, mini‐fyke nets, standard fyke nets, large and small hoop nets, and trawls) in multiple habitat strata (i.e., main channel border, side channel border, and backwater) during three, 6‐week sampling periods (June 10–October 31). Across these four rivers, Spotted Bass collections reported here were collected by the LTEF standardized random and fixed direct‐current boat electrofishing sampling on the IWW, UMR, Ohio, and Wabash rivers (see Fritts et al., 2017), LTRM multigear, multistrata sampling on Pool 26 of the UMR and the La Grange Pool of the IWW (see Ratcliff et al., 2014), and IDNR's rivers and streams program's alternating‐ and direct‐current boat electrofishing and electric seine surveys on the Illinois portions of the Upper Mississippi, Ohio, and Wabash rivers.

## 3 RESULTS

Collectively, the IDNR, LTRM element, and LTEF program have documented 9593 Spotted Bass collections from 1963 to 2021 in the Illinois waters of these four large rivers, with two individuals being collected from the IWW, 1047 individuals from the UMR, 2299 individuals from the Ohio River, and 6245 individuals from the Wabash River. The greatest number of Spotted Bass collections occurred in their native range in Illinois, the Ohio and Wabash rivers (Figure 2). Within the five LTEF sampling reaches of the unimpounded lower Wabash River, the greatest number of Spotted Bass collections occurred in the three middle reaches (Figure 2). On the Ohio River, LTEF data showed Spotted Bass collections were the greatest upstream in the Smithland Pool and then decreased in number downstream to the Upper Mississippi and Ohio rivers confluence (Figure 2). The majority of the UMR Spotted Bass collections from 1972 to 2021 were in the Kaskaskia and Chain of Rocks open‐river reaches between the Melvin Price Lock and Dam (322.7 river km) and the Upper Mississippi and Ohio rivers confluence (Figure 2). However, there have been 10 Spotted Bass reported above the Melvin Price Lock and Dam. These collections include two Spotted Bass from Pool 25 (IDNR 1980 and LTEF 2012), and eight Spotted Bass from Pool 26, two in 1989, one in 2017, and six in 2019 (IDNR; Metzke et al., 2022).

In 2020, LTRM collected its first records of Spotted Bass in Pool 26 of the UMR. The first record was collected on June 24, 2020, during daytime electrofishing along a connected backwater shoreline near Lock and Dam 25, upstream of the UMR and IWW's confluence (Table 1; Figure 2). The second record was collected on August 10, 2020, in a mini‐fyke in the Eagle Nest Island side channel downstream of the UMR and IWW's confluence (Table 1; Figure 2). No Spotted Bass were recorded in 2021 in Pool 26 of the UMR by LTRM, LTEF, or the IDNR.

**TABLE 1:** Sampling location, gear, program, and fish size (total length and weight) of new Spotted Bass *Micropterus punctulatus* records collected from the Illinois Waterway and Upper Mississippi River.

River	Date	River pool	River kilometer	Latitude/longitude	Sampling gear	Length (mm)	Weight (g)	Sampling program
Illinois Waterway	2‐Sep‐11	La Grange	238.2	40°33′19″ N 89°43′14″ W	Electrofishing	351	621	LTEF
	1‐Oct‐21	Peoria	290.0	40°54′28″ N 89°29′9″ W	Electrofishing	247	199	LTEF
Upper Mississippi River	24‐Jun‐20	26	388.8	39°0′9″ N 90°41′33″ W	Electrofishing	270^a^	–	LTRM
	10‐Aug‐20	26	338.4	38°55′55″ N 90°18′1″ W	Mini‐fyke Net	60^a^	–	LTRM

^a^ Fish length recorded in 10 mm length bins.

Two records of Spotted Bass have been collected in the IWW by the LTEF program, and to our knowledge, these records are the first reported collections from the main stem of the IWW (Figure 2). Five other Spotted Bass records have been documented in smaller rivers and streams surrounding the IWW's Alton and La Grange pools (Metzke et al., 2022; Figure 2). The first collection of Spotted Bass in the IWW was on September 2, 2011, in the Turkey Island side channel of the La Grange Pool (Table 1; Figure 2). This collection was upstream of the mouth of the Mackinaw River (40°33′8.28″ N, 89°43′48″ W), a tributary to the IWW, where one Spotted Bass was collected in 2009 (Fuller et al., 2021; Figure 2). At that time, the closest known population of Spotted Bass connected to the IWW occurred in the Chain of Rocks open‐river reach of the UMR (Figure 2). The second and most recent collection of a Spotted Bass in the IWW occurred on October 1, 2021, in the main channel of the Peoria Pool near Chillicothe, Illinois (Table 1; Figure 2). This record was the first IWW main channel collection of Spotted Bass and was 32 river miles and one navigation pool upstream from the 2011 record. The 2021 Spotted Bass was given to the Illinois Natural History Survey (INHS) Fish Collection in Champaign, Illinois, as a voucher specimen (INHS 153135).

## 4 DISCUSSION

Over the past decade, Illinois has observed an increase in Spotted Bass collections outside of their native range in the UMR and IWW. Tracking collections of Spotted Bass outside of their native range in the large rivers of Illinois is key to increasing our knowledge of Spotted Bass movement and habitat use in large rivers and also informing fisheries management decisions, given currently unknown effects of non‐native Spotted Bass on native riverine fishes. Here, we report the current collections of Spotted Bass in the four large rivers of Illinois using three different fisheries monitoring programs and highlight the first reported collections in the IWW to track the current distribution and potential future population expansion of Spotted Bass in Illinois.

Spotted Bass movement and habitat use within and between streams and rivers is variable within individual lotic systems and can differ based on seasonality, individuals (e.g., age and size), and habitat characteristics (e.g., water depth, velocity, woody structures, and aquatic vegetation; Abell et al., 2018; Baebler, 2018; Edge et al., 2020; Goclowski et al., 2013; Lewis & Elder, 1953)—thus, determining movement and habitat use for individual systems is ideal for management. Prior studies have focused on Spotted Bass movement patterns in streams (Goclowski et al., 2013; Lewis & Elder, 1953), but limited literature exists for large rivers (Baebler, 2018) and between large rivers and their tributaries (Abell et al., 2018). Differential seasonal movement rates (Baebler, 2018) and seasonal selection of habitat (water velocity, water depth, and habitat preference; Edge et al., 2020) have been documented in large rivers. Additional collections of Spotted Bass in the IWW and Pools 26 and 25 of the UMR would be useful for studying their distribution, habitat use, and impacts on native species in these areas through the use of telemetry, external tagging (e.g., Floy tag), genetic sampling, and otolith microchemistry. These tools could be used to reconstruct natal origin of Spotted Bass from the IWW and UMR to potentially understand whether source populations are from natural expansion or intentional introduction(s) and determine seasonal movement and habitat use that could impact recreationally valuable native fishes' (Smallmouth Bass *M. dolomieu* and Largemouth Bass *M. salmoides*) population dynamics, distribution, and hybridization (e.g., Godbout et al., 2009; Long & Fisher, 2005).

The IWW and UMR are intensively sampled and have been for decades (e.g., Fritts et al., 2017), by multiple long‐term monitoring programs (i.e., LTEF, IDNR, LTRM, Multi‐Agency Monitoring [MAM]), research, and removal projects (e.g., IDNR invasive bigheaded carps [Silver Carp *Hypophthalmichthys molitrix* and Bighead Carp *H. nobilis*] removal); therefore, they have the capability of capturing range expansion in real time. In the IWW, these programs have tracked past expansions of multiple native and non‐native fish species. For example, they have documented the establishment and expansion of non‐native, invasive bigheaded carps in the IWW, which were first documented in the 1990s and became established in the La Grange Pool in 2000 (Sass et al., 2010; Figure 2). Currently, adult bigheaded carps can be collected from Alton Pool to Dresden Island Pool in the IWW (e.g., Whitten & Gibson‐Reinemer, 2018; Figure 2). Further, LTEF has tracked the increase in the proportion of native sport fishes in the upper pools of the IWW (Starved Rock, Marseilles, and Dresden Island pools) in the latter half of the twentieth century following the Clean Water Act of 1972 (Gibson‐Reinemer et al., 2017). Thus, if an established population of Spotted Bass existed in the IWW, it is unlikely that it would go undetected.

Long‐term monitoring programs, research projects, and reporting of new species occurrences, or lack thereof, help track range expansions or contractions of native and non‐native fishes. Collaborative projects and data sharing among agencies, and across large geographical areas, are beneficial in noting population changes in native and non‐native fish species. Using this information to educate managers and the public of changes in species ranges and accordingly inform policy is crucial in managing the distribution of native and non‐native fishes. The lack of this knowledge would stall future improvements in large river fisheries management, particularly given the current commercial and recreational use of these interconnected freshwater systems.

## AUTHOR CONTRIBUTIONS

**Andrya L. Whitten:** Conceptualization (equal); writing – original draft (lead); writing – review and editing (lead). **Brandon S. Harris:** Conceptualization (equal); writing – review and editing (supporting). **Jason A. DeBoer:** Conceptualization (equal); writing – review and editing (supporting). **Nerissa N. McClelland:** Resources (supporting); writing – review and editing (supporting). **James T. Lamer:** Funding acquisition (lead); writing – review and editing (supporting).

## CONFLICT OF INTEREST

The authors declare no conflict of interest.

## Data Availability

The data that support the findings of this study are in part openly available from the Upper Mississippi River Restoration Program's Long‐Term Resource Monitoring (LTRM) element at https://umesc.usgs.gov/data_library/fisheries/fish1_query.shtml and upon request from the corresponding author.
